# Heteroepitaxial GaAs thin-films on flexible, large-area, single-crystal-like substrates for wide-ranging optoelectronic applications

**DOI:** 10.1038/s41598-024-59686-0

**Published:** 2024-05-02

**Authors:** Gokul Radhakrishnan, Kyunghoon Kim, Ravi Droopad, Amit Goyal

**Affiliations:** 1 TapeSolar Inc., Knoxville, TN 37922 USA; 2 Ingram School of Engineering, Texas State University, San Marcos, TX 78666 USA; 3 Laboratory for Heteroepitaxial Growth of Functional Materials & Devices, Department of Chemical & Biological Engineering, State University of New York at Buffalo, Buffalo, NY USA

**Keywords:** Engineering, Materials science

## Abstract

Recent advances in semiconductor based electronic devices can be attributed to the technological demands of ever increasing, application specific markets. These rapidly evolving markets for devices such as displays, wireless communication, photovoltaics, medical devices, etc. are demanding electronic devices that are increasingly thinner, smaller, lighter and flexible. High-quality, III-V epitaxial thin-films deposited on single-crystal substrates have yielded extremely high-performance, but are extremely expensive and rigid. Here we demonstrate heteroepitaxial deposition of GaAs thin-films on large-grained, single-crystal-like, biaxially-aligned, flexible, metallic substrates. We use molecular beam epitaxy (MBE) for the controlled growth of high quality GaAs layers on lattice matched Ge capped, flexible metal substrates. The structural, optical, interfacial and electrical characteristics and properties of the heteroepitaxial GaAs layers are analyzed and discussed. The results show that heteroepitaxial GaAs layers with good crystalline and optoelectronic properties can be realized for flexible, III-V based semiconductor devices. III-V materials integrated on large-grained, single-crystal-like, flexible, metallic substrates offer a potential route towards fabrication of large-area, high-performance electronic devices.

## Introduction

Rapid development in performance metrics of electronic devices has enabled their presence in all aspects of life. The ever increasing, application specific technological demands on these electronic devices have pushed the semiconductor industry to develop thinner, smaller, lighter, more robust devices for broad-ranging markets such as displays, wireless communication, photovoltaics, medical devices, etc. There is demand for thin and flexible wearables and sensors, etc., that can conform to curved surfaces and are light-weight. While thin-film semiconductor devices on polycrystalline or polymer substrates are low-cost alternatives, their performance is very low due to numerous defects and grain boundaries and a lack of precise control over the growth of grains and the semiconductor films. Single-crystal semiconductor films on high-quality single-crystal substrates are indispensable for delivering unparalleled high performance. For applications requiring high performance, current technology relies on these epitaxial-growth ready single crystal substrates that are extremely expensive, available only in small sizes and are rigid. A route to developing robust, flexible, high performance electronic devices could potentially be the successful growth of heteroepitaxial GaAs films on large-area, single-crystal-like, flexible, cost-effective artificial substrates.

The advanced physical properties of III-V (GaAs, etc.) based semiconductor thin films over silicon has led to development of high-performance electronics. Their high electron mobility has led to advanced RF electronic/optoelectronic devices and high absorption coefficient in turn has aided in the development of state-of-the-art, ultra-high, efficient photovoltaic devices^1–8^. High absorption coefficient of III-V based materials means a thin solar cell (2-5 µm) is sufficient to achieve high power^9^. Hence, the use of thick substrates is not necessary and this reduces the mass specific power which is important for space and weight specific applications. The advantage of high performance cannot be fully realized due to the associated high substrate cost. Addressing the technical challenges, while following the proven manufacturing methods of conventional III-V based devices has been difficult. The epitaxial lift-off (ELO) method and direct wafer bonding where the active layers are chemically separated from the host substrate have shown impressive results^10–12^. Though these approaches address both cost – by substrate reuse (over hundreds of times) and weight—as epitaxial thin films, commercial large-scale implementation still remains a significant challenge since reuse of the expensive substrates beyond a few times results in significant decrease in performance^13^. There is significant demand in terrestrial applications (roof tiles), off-grid locations, satellites and unmanned aerial vehicles (UAVs) for power generated using lightweight, high-efficient, flexible solar cells. Currently there is no technology that can offer the potential for large-scale production of high-performance, III-V devices on cheap or cost-effective substrates that is light-weight, has high power density and is flexible.

In this work, we report the fabrication of heteroepitaxial, triaxially-aligned, GaAs thin films on a single-crystal-like, flexible metal/alloy substrates (Ni-based) fabricated via thermomechanical processing and heteroepitaxial growth^14–16^. The technique uses well established, industrially scalable, thermomechanical processes to impart a strong biaxial texture to a base metal. The process involves successive mechanical rolling to a high deformation (> 99%) followed by high temperature annealing. The annealing process leads to the formation of large, single-crystal grains through recrystallization^14–16^. In addition, all the large grains are triaxially textured, wherein all three of the crystallographic axes of all the grains are aligned to within a few degrees, with the whole assemblage similar to a large single-crystal with a mosaic of a few degrees. Heteroepitaxial oxide buffer layers YSZ, Y_2_O_3_, CeO_2_ respectively are deposited to provide an excellent crystalline template, chemical barrier and lattice matching for subsequent semiconductor thin film deposition. A perfectly lattice matched layer of high-quality epitaxial Ge thin film is grown on CeO_2_ and serves as a template for the growth of heteroepitaxial GaAs films. In this work, we report on the heteroepitaxial deposition of GaAs on the large-area, single-crystal-like, flexible, light-weight, single-crystal-like substrates using Molecular Beam Epitaxy (MBE). The structural, optical and electronic properties of the GaAs films are analyzed, to explore the possibility of using this as a virtual flexible substrate for large-scale fabrication of high-performance electronic devices.

## Results and discussions

**Figure 1:**
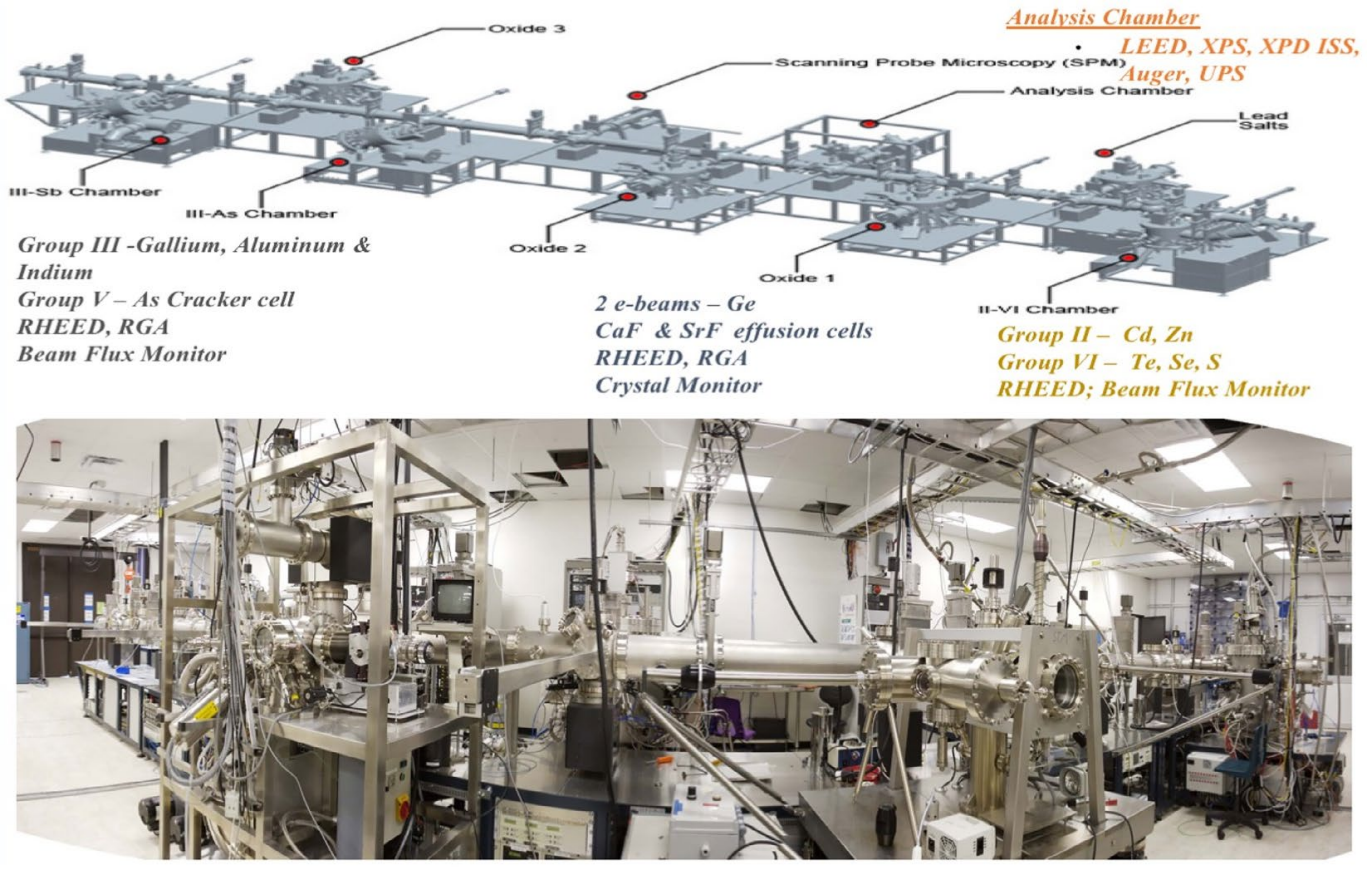
Schematic of system used to deposit heteroepitaxial layers (top) and picture of actual facility (bottom).

The deposition of GaAs was carried out in a DCA M450 III-V based MBE chamber which is part of an ultra-high vacuum, multi-chamber system. All chambers are inter-connected through ultra-high vacuum buffer chambers (< 5 X10^–10^ torr). A schematic and picture of the experimental setup used for this work is shown below Fig. 1.

This arrangement allows for the growth and analysis of different materials *in-situ* without exposure to atmosphere. First Ge was deposited on metal/buffer stack using electron-beam evaporation in a separate but interconnected MBE chamber. The deposition conditions and other parameters involved for the growth of defect-free, single-crystal-like Ge films on flexible alloy substrates is discussed in detail in our previous work^17^. Ge films were heteroepitaxially grown on flexible, large-area, light-weight, single-crystal-like, CeO_2_-terminated, heteroepitaxial buffer stack on the metallic substrate using electron beam evaporation in vacuum in the temperature range of 600 ~ 700 °C with a thickness of 2–3 μm. The CeO_2_ layer serves as a highly compliant layer that modulates its lattice parameter to attain excellent lattice-matching to the heteroepitaxial Ge layer. The Ge film is essentially single-crystal-like with a very low defect density.

**Figure 2:**
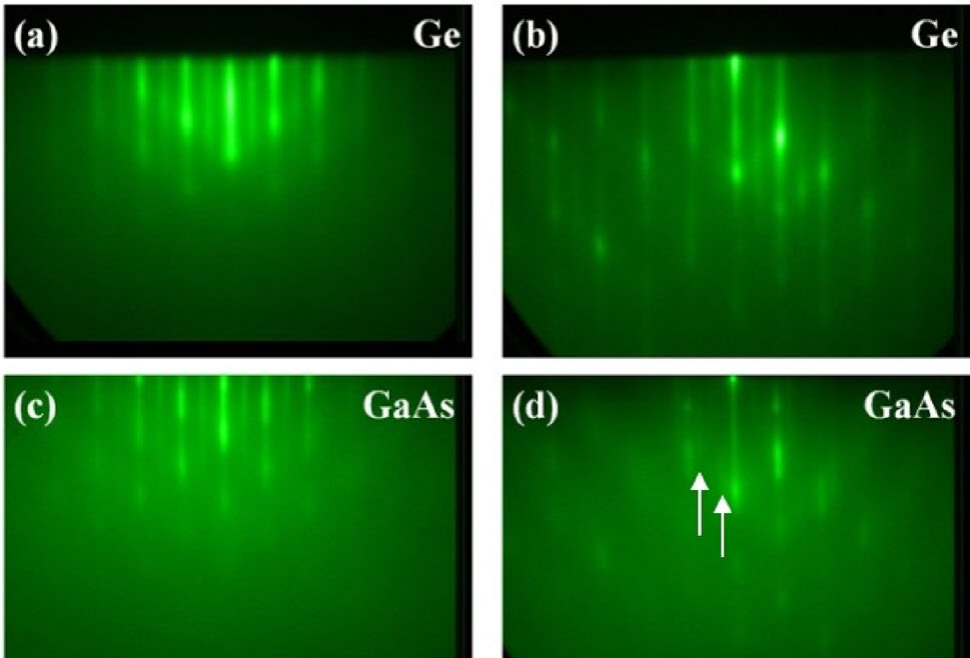
(**a**) and (**b**) shows the RHEED pattern taken on Ge surface, where there is clear streaky two-fold symmetry at the [110] and [$\overline{1 }$ 10] azimuths respectively, (**c**) and (**d**) shows the RHEED pattern taken on the GaAs surface, where there is clear streaky two fold symmetry at the [110] and four fold symmetry at the [$\overline{1 }$ 10] azimuths respectively. The arrows in (**d**) shows the faint ¼ and ¾ features.

Samples with 3 μm thick Ge films deposited on metal/buffer stack were transferred through the UHV buffer line and loaded into the M450 (III-V) based MBE chamber. In order to ensure controlled application of pure Ga and/or As for good nucleation, prior to loading the samples the As background pressure was kept at 10^–10^ torr or lower. This was achieved by keeping the shutter and valve of the As cracker cell fully closed along with a 12 h wait period between each growth runs. To monitor the sample surface and control the growth conditions, in-situ tools such as infrared pyrometer, manipulator thermocouple and Reflection High Energy Electron Diffraction (RHEED) were used. During the growth process the RHEED patterns were monitored to observe the surface structure of the starting layer and the GaAs film. The Ge layer showed a clear (2 × 2) streaky RHEED pattern at 350 °C before the start of the low temperature migration-enhanced-epitaxy (MEE). This RHEED pattern is shown in Fig. 2a,b where there is a clear streaky two-fold symmetry in both the [110] and [ḹ10] azimuths respectively. This indicates the presence of steps on the surface of Ge with single monolayer heights. On some terraces the dimer rows are parallel to the step edges while on the adjacent steps the dimer rows are perpendicular to the step edges.

In order to increase the density of double atomic surface step, the sample was subject to an *in-situ* thermal annealing at 640 °C^18^. The sample was then cooled to initiate the migration enhanced epitaxy (MEE) sequence. The MEE was done by the alternate exposure of As and Ga adatoms on the Ge surface. During the Ga exposure both the shutter and valve of the As cracker cell were closed to ensure complete coverage by Ga atoms. The low temperature MEE (~ 250 °C) sequence consisted of 10 to 20 cycles with the growth rate ranging between 0.05 to 0.20 µm/h. As and Ga adatom exposure times was calculated for one monolayer growth for every cycle. After 10 monolayer the MEE sequence was stopped, with an As overpressure maintained. Following this a 0.25 µm thick low temperature GaAs buffer was grown by opening both the shutters. Thereafter the substrate temperature was increased to 590 °C for growing a 1–1.5 µm thick undoped GaAs layer at 1 µm/h at an As_2_/Ga BEP (beam equivalent pressure) ratio of ~ 25.

The microstructural quality of the GaAs film grown on Ge-terminated metallic templates was characterized using high-resolution X-ray diffraction (XRD), electron backscatter Kikuchi diffraction (EBKD) and orientation imaging microscopy (OIM). The surface, crystal and optical quality of the GaAs layer was investigated using scanning electron microscope (SEM), high resolution TEM (HRTEM) and room-temperature photoluminescence (PL) measurements. The electronic properties were characterized via Hall measurements using a BioRad Accent HL5500 Hall system with a magnetic field of 0.325 T. Carrier mobility, bulk carrier concentration and resistivity were measured using this system and all measurements were done at room temperature on a square geometry with four indium ohmic contacts placed at the edges of the 10 mm square samples. A current of 0.1 mA was used during the measurement and the results (mobility and carrier concentration) were displayed on the screen.

During the MBE growth, RHEED patterns were monitored to observe the surface structure of the starting layer and the GaAs film. The Ge layer showed clear (2 × 2) streaky RHEED pattern at 350 °C before the start of the low temperature MEE suggesting the surface consist of monolayer steps. The RHEED pattern is shown in Fig. 2a,b where there is clear streaky two-fold symmetry at the [110] and [1 $\overline{1 }$ 0] azimuths respectively.

The (100) surface of Ge consists of dimers resulting in a (2 × 1) surface reconstruction. However the terraces of single step heights would have dimers perpendicular to each other resulting in a (2 × 2) surface reconstruction^19,20^ as shown in Fig. 2a,b. Thus, each individual step terrace should be present an ideal surface condition for the nucleation of polar GaAs over the nonpolar Ge, so as to prevent and or mitigate the formation of anti-phase domains (APD)^21–23^ under optimized deposition conditions. It is expected the within each grain the Ge surface is step-free providing the conditions for APD free GaAs growth.

Throughout the MEE sequence the RHEED reconstruction remained streaky. At the end of the MEE a clear GaAs c(4 × 4) RHEED pattern reconstruction is seen confirming smooth two dimensional surfaces and the absence of three dimensional island growths. At the end of growth process the GaAs surface showed an As stabilized (2 × 4) streaky RHEED pattern at 590 °C. The final RHEED pattern is shown in Fig. 2c,d where there is clear streaky two-fold symmetry at the [110] and four-fold symmetry at the [$\overline{1 }$ 10] azimuths respectively shown by the faint ¼ order features. This surface structure represents the well-known single domain As stabilized GaAs (001) 2 × 4 reconstruction.

**Figure 3:**
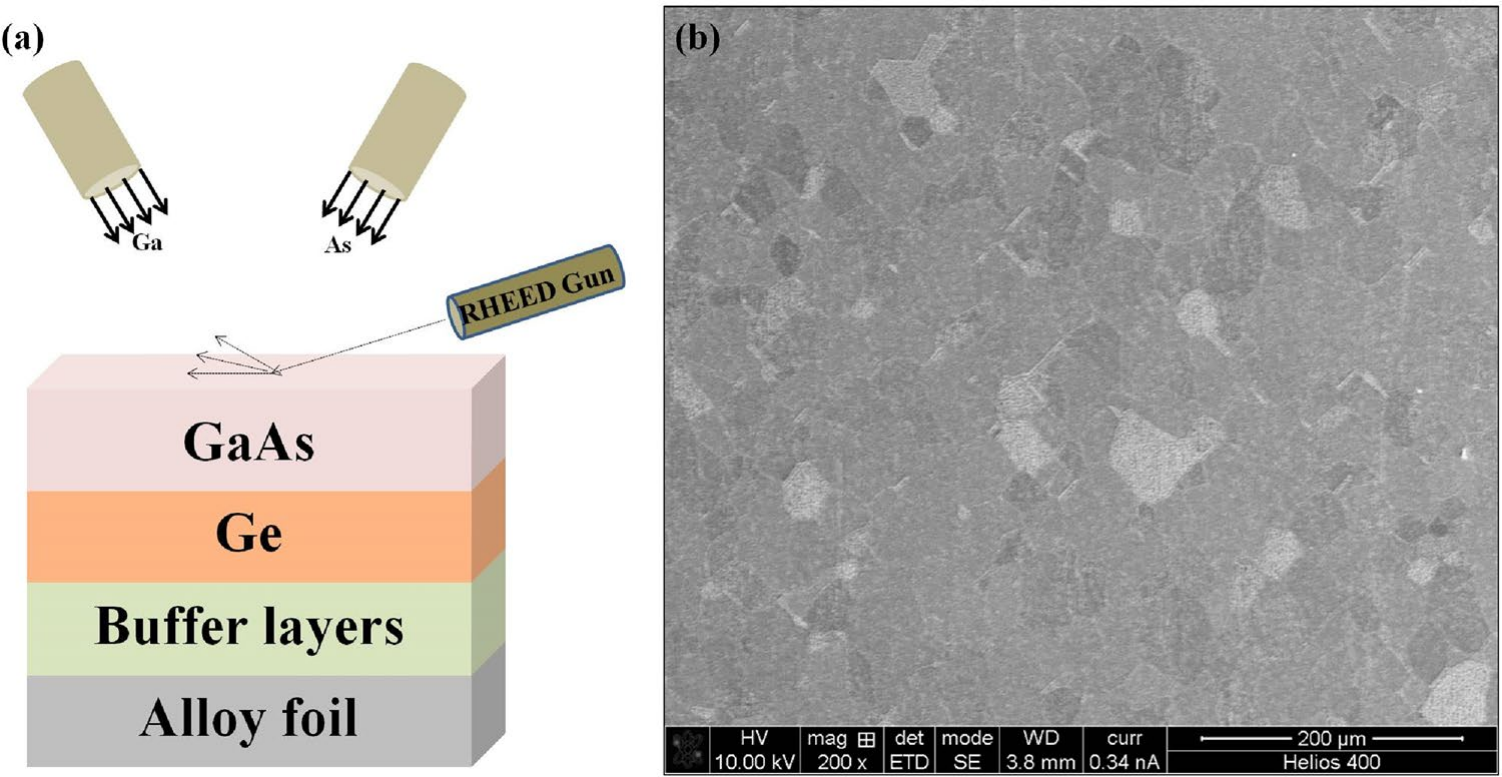
(**a**) Schematic representation of the MBE grown GaAs and Ge film on the buffered metal substrate. (**b**) Scanning electron micrograph (SEM) of the GaAs surface.

It should be noted here that domains on all GaAs grains are not oriented in the same direction. When the electron beam is scanned over the GaAs surface from one grain to another, the RHEED reconstruction can for some grains change from 2 × to and 4 × i.e. confirming the presence of single domains within the grains but domains in some grains can change orientation by 90°. This may have to do with detailed orientation of some grains, including the out-of-plane and in-plane orientation, resulting in certain miscut surfaces. Figure 3a shows a schematic of the MBE grown GaAs and Ge film on the buffered metal substrate and Fig. 3b shows the top view SEM analysis of the post growth GaAs film. The SEM picture shows well-defined individual grain structures ranging in size from ~ 50–100 microns. These grains were analyzed individually and collectively (thousands) to determine their crystalline quality.

**Figure 4:**
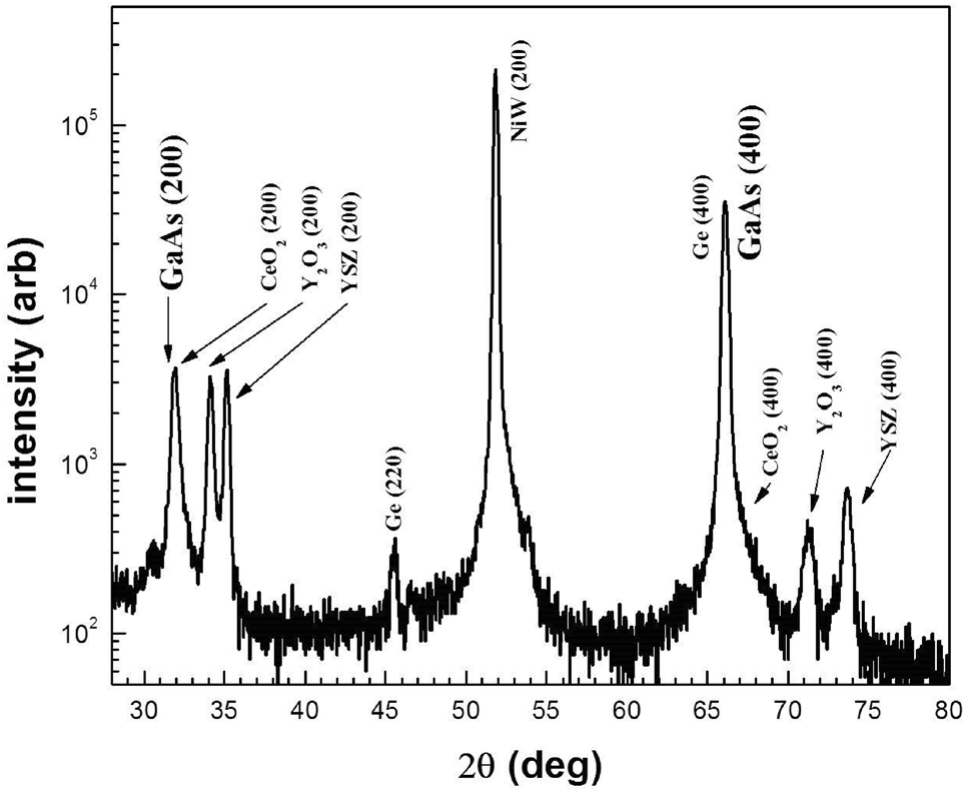
θ–2θ X-ray diffraction scan of the GaAs film.

High-angle X-ray diffraction (HAXRD), theta-2theta and rocking curve scans were done to analyze the crystallographic orientation and complete structural quality of the GaAs epitaxial layer. Figure 4 shows the X-ray diffraction θ-2θ scan of the GaAs film on the Ge/Buffer layers/Ni-W metal substrate along the entire 2 theta angle. Ni-W XRD pattern of (200) orientation appears at 51.6°. XRD patterns of (200) oriented buffer layers CeO_2_, Y_2_O_3_ and YSZ appear at 2θ values of 33.0°, 33.9° and 34.9° respectively.

XRD patterns of (400) oriented buffer layers CeO_2_, Y_2_O_3_ and YSZ appear on the 2θ scale at 69.5°, 71.1° and 73.6° respectively. The scan shows a major Ge (400) peak at around 66.0° and minor peaks at 45.6° from Ge (220) orientation.

**Figure 5:**
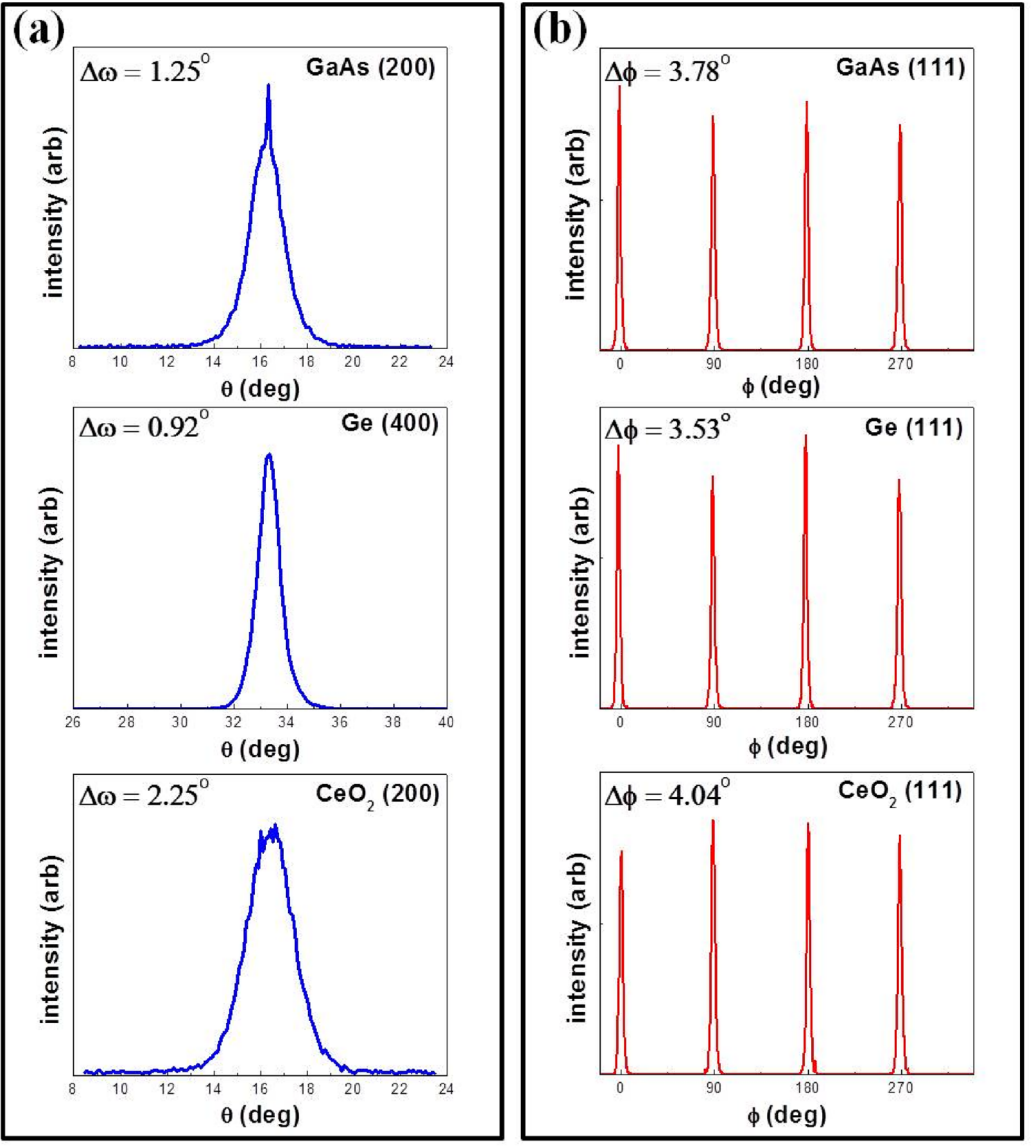
(**a**) High angle XRD scans demonstrating the orientation of the out-of-plane axis when rocking about perpendicular directions (**b**) XRD scans demonstrating the orientation of the in-plane texture, that shows that all the heteroepitaxial, cube-on-cube oriented grains in the GaAs and Ge layer are aligned within ~ 4°.

Finally, the X-ray θ-2θ scan shows strong and dominant GaAs (002) and (004) peaks at 32.0° and 66.0° respectively, providing initial evidence for high-quality, single-crystal-like, heteroepitaxial GaAs thin film. Figure 5a shows HAXRD scans demonstrating the orientation of the *out-of-plane* axis when rocking about the perpendicular directions. It can be seen that the c-axis of all the grains in the GaAs and Ge layers are aligned within ~ 1°.

Figure 5b shows HAXRD scans demonstrating the orientation of the *in-plane* texture that shows that all the heteroepitaxial, cube-on-cube oriented grains in the GaAs and Ge layer are aligned within ~ 4°. Such a GaAs surface is essentially single-crystal-like with a grain size of about 100 microns. The mosaic in the rocking-curve and the phi-scan is due to slight orientation differences between individual grains in this substrate having an average size of 100 microns. Within a grain, the GaAs is essentially a single-crystal. Using this technique, it is also possible to control the grain sizes from several centimeters to an inch^14–17^.

**Figure 6:**
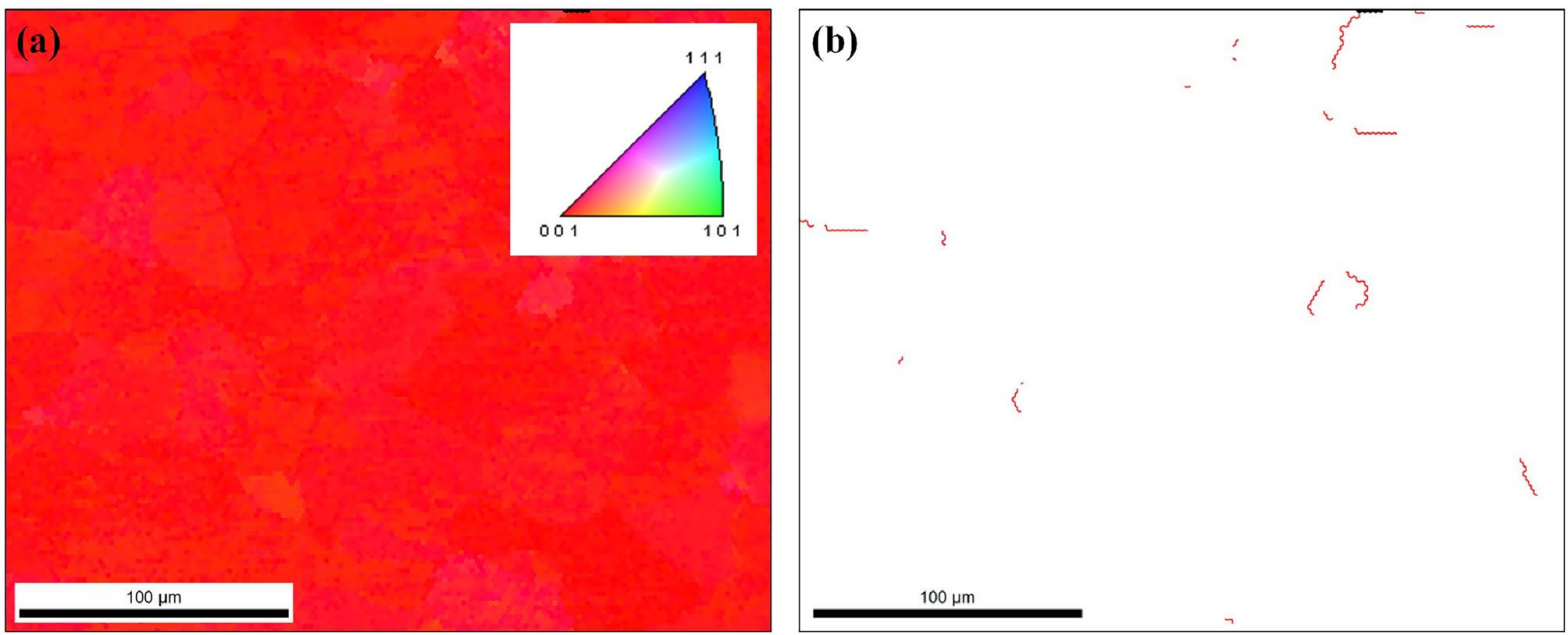
(**a**) Point-to-point orientation data taken from the GaAs surface (**b**) all grain boundaries are shown where they are present. The red lines show the grain boundaries with a misorientation angle greater than 2°.

Figure 6a shows point-to-point orientation data taken from the GaAs surface. This data is generated using electron backscatter Kikuchi diffraction (EBKD) done on a hexagonal grid at a spacing of 0.5 microns. By taking such EBKD patterns on a hexagonal grid, the 100, 000 or so electron diffraction patterns taken and indexed were then used to generate the color-coded microstructural image shown in this figure. This orientation image micrograph (OIM) is created using the orientation data. The inset of this OIM image shows the criteria used for coloring. As can be seen, effectively the entire 0.25 mm × 0.15 mm region is essentially of the same orientation. On the right in Fig. 6b all grain boundaries are shown where they are present. The red lines show the grain boundaries with a misorientation angle greater than 2°.

**Figure 7:**
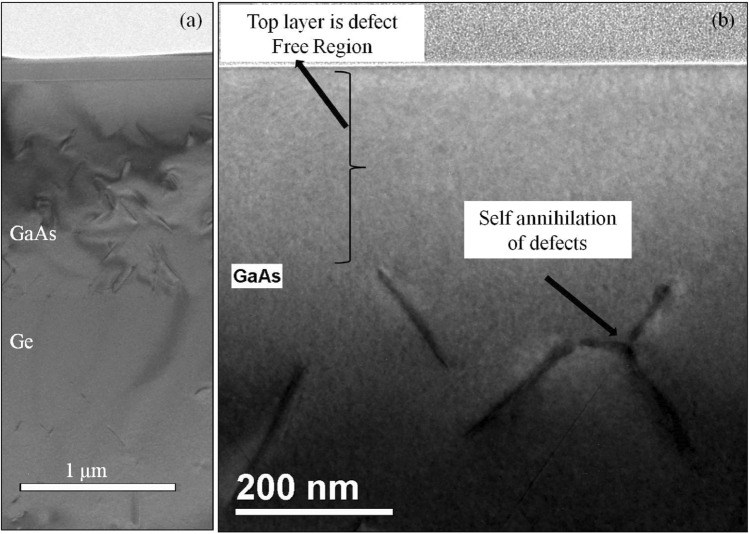
(**a**) shows cross-sectional transmission electron microscopy (XTEM) images of the sample with epitaxial GaAs layer. (**b**) shows a higher-magnification image of the top area of the GaAs layer.

To access the crystal quality of the GaAs film grown on these single-crystal-like substrates, high resolution TEM analysis was done. The TEM imaging was done using a JOEL-2010 microscope at 200 keV. The sample was prepared for cross-sectional analysis using a focused ion beam system (FIB). Figure 7a shows the dark-field cross-sectional transmission electron microscopy (XTEM) images of the sample with the epitaxial GaAs layer on Ge/metal buffer stack. There is evidence here showing the presence of stacking faults and dislocation-like defects. These defects are predominantly confined to the (111) plane and such defect formation is evidence for the presence of antiphase boundaries (APBs)^24,25^.

The growth of III-V semiconductors on group IV semiconductors typically result on APDs, threading dislocations (TDs) and possible stacking faults. APDs results from nucleation of the polar on non-polar growth (GaAs on Ge) whereas TDs results from lattice mismatch or differences in the coefficient of thermal expansion of the 2 materials. APDs can also originate at step edges and propagate upwards or 45°. These APDs and stacking faults or dislocations depending on the point of origin travel along the (111) planes eventually intersecting with each other leading to self-annihilation of the twins. Figure 7b is a higher magnification image that clearly shows the self-annihilation of APDs. After a certain thickness of epitaxial GaAs the defect density reduce significantly and the top layer of GaAs has significantly lower defects. There is also a possibility that defects originated from the Ge/oxide interface may also propagate into the GaAs layer. Nevertheless, this surface will serve as a template for the growth of good quality GaAs layers for device fabrication.

**Figure 8:**
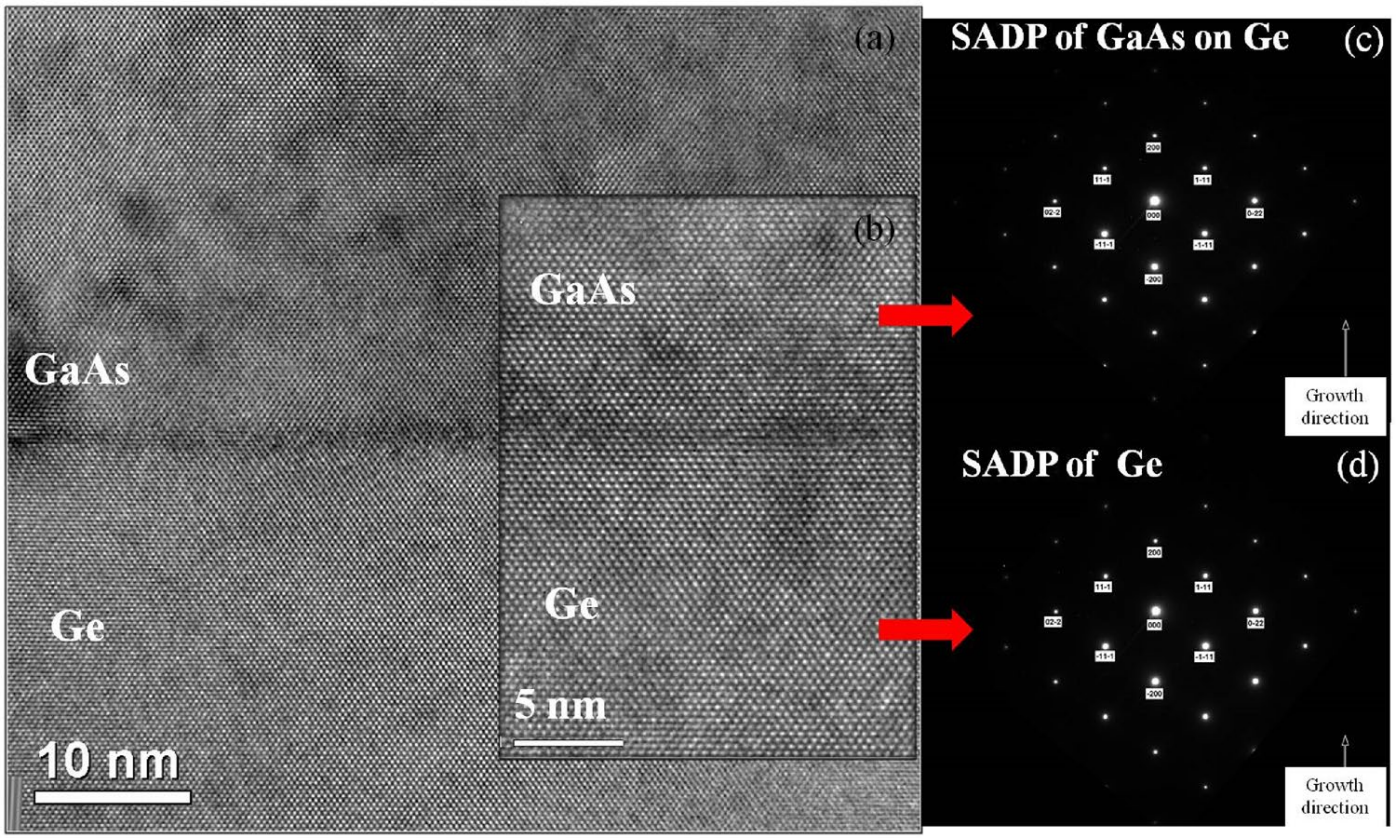
(**a**) and (**b**) shows the HRTEM image of the interface between Ge and GaAs at increasing magnifications and (**c**) shows the selected area electron diffraction patterns (SAED) of GaAs and (d) shows the selected area electron diffraction patterns (SAED) of Ge.

Figure 8a,b shows the HRTEM image of the interface between Ge and GaAs at increasing magnifications and Fig. 8c,d show the selected area electron diffraction patterns of GaAs and Ge respectively. These diffraction patterns are characteristically spotty confirming the epitaxial and high-quality, single-crystal-like nature of both Ge and GaAs layers. From the TEM images the total defect density in the GaAs epilayer is estimated to be in the low 10^8^ cm^−2^. By reducing the MEE sequence temperature and by carefully controlling the As_2_/Ga BEP ratios the defect density can be reduced to the low 10^7^ cm^−2^ range, which can directly influence the performance of devices fabricated on these epilayers^26^.

**Figure 9:**
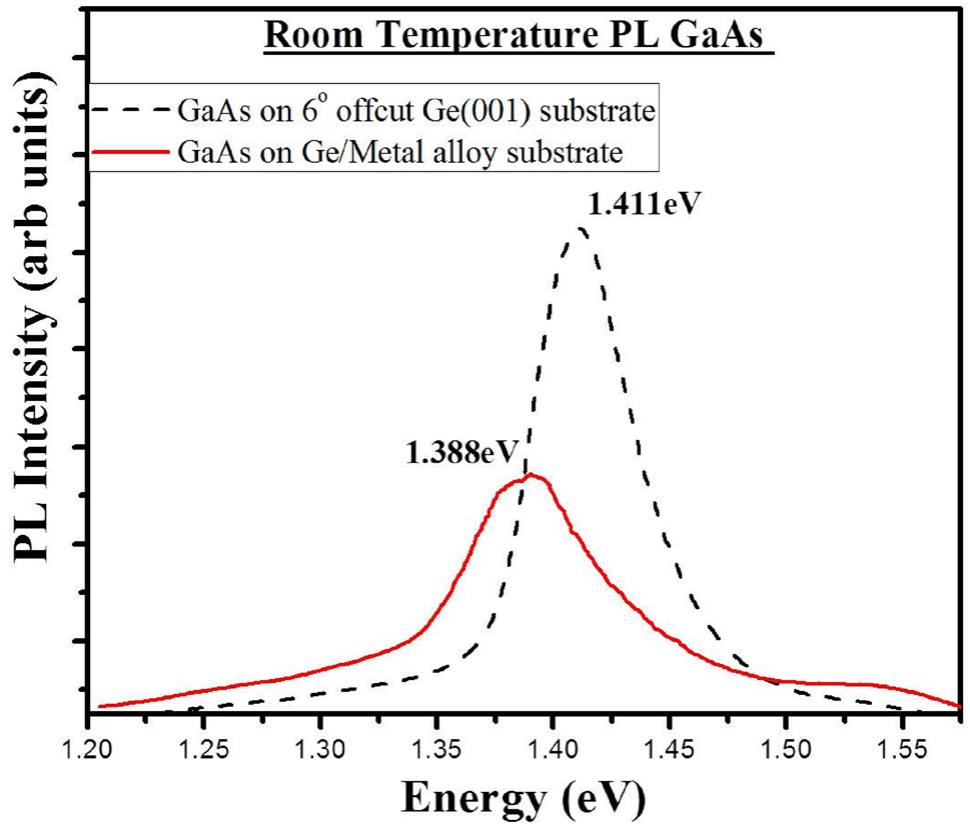
Room temperature PL spectra of the 1 µm undoped GaAs layer on Ge/metal alloy stack (red bold line) and single crystal 6° offcut Ge (001) substrate (dotted line).

Room temperature PL spectroscopy was carried out to investigate the optical properties of the GaAs epitaxial film. A 514 nm argon laser (200mW) with laser line filters was used, along with a SpectraPro 300i monochromator to excite a 30 µm sample area. To compare the luminescence data 1 µm undoped GaAs was grown epitaxially on a 6° offcut (001) oriented single crystal Ge substrate. The growth conditions for this deposition involved the same MEE sequence for the thin nucleation layer followed by low temperature buffer and high temperature deposition of the rest of the layer. Figure 9 shows the room temperature PL spectra of the 1 µm undoped GaAs layer on Ge/metal alloy stack (red bold line) and single crystal 6° offcut Ge (001) substrate (dotted line).

A PL peak at 1.388 eV is observed for the GaAs layer on Ge/metal alloy stack. The bulk GaAs on Ge single crystal substrate shows a PL peak at 1.411 eV which is the typical band to band transition for GaAs. The full width half max (FWHM) of the solid PL peak is 0.085 eV with is comparable to the FWHM of the dotted PL peak of 0.064 eV. This shows the epitaxial GaAs on Ge/Metal alloy stack is of reasonable optical quality. The red bold PL peak has reduced peak intensity and is red shifted by 0.02 eV from the dotted bulk PL peak, possibly due to internal strain. The PL spectra can also be affected by APBs and near band edge point defects or impurities in the GaAs layer.

Hall Effect measurements were carried out to determine the carrier mobility of the epitaxial GaAs film. All measurements were done at room temperature using Accent HL5500 Hall system. All growths were done using MBE (Molecular beam epitaxy). The growth conditions such as GaAs film growth temperature, as and Ga beam equivalent pressure, pre-annealing and post-annealing temperatures^27,28^, were changed in order to probe their effects on carrier mobility. Reducing the presence of as atoms in the MBE chamber before the start of the MEE sequence is key for 2D growth of GaAs. So, prior to loading a sample the as background pressure was kept at 10^–10^ torr or lower by keeping the shutter and valve of the as cracker cell fully closed, along with a 12 h pump down time between growth runs. First a 1 µm thick undoped GaAs thin film was grown on Ge/Flexible Alloy stack. This layer will act as a barrier to the underlying Ge layer during Hall measurements. Following that a 1 µm thick Si doped GaAs layer with a carrier concentration of ~ 2 × 10^18^ was grown. Hall measurement done on this film yielded an electron mobility of 346 (cm^2^V^−1^ s^−1^). High temperature annealing was explored on another sample by annealing the GaAs film post growth at 750 °C for 15 min under high as overpressure. This improved mobility and yielded a peak electron mobility of 459 cm^2^V^−1^ s^−1^ which is the highest reported value for a heteroepitaxial GaAs film on a single-crystal-like, polycrystalline substrate that can be fabricated continuously in large-areas via roll-to-roll fabrication. Mobilities for *single-crystal* GaAs films at the same doping level are around ~ 2000 cm^2^V^−1^ s^−1^ as shown in Fig. 2 of Sotoodeh et al.^29^, and so the mobility of these heteroepitaxial films are a factor of ~ 4 less than that of single-crystal GaAs. Mobilities of amorphous or randomly oriented, non-heteroepitaxial GaAs, Ge and Si films are *orders of magnitude lower* that of single-crystal GaAs as well as the films reported here.

## Conclusions

Heteroepitaxial deposition of GaAs layers on large-area, flexible, single-crystal-like, metallic substrates was demonstrated. Within each substrate grain, the GaAs film is effectively single-crystal. Orientation image microscopy images show that across an entire 0.25 mm × 0.15 mm region containing multiple grains, the GaAs layer is essentially of the same orientation with only a few grain boundaries greater than a couple of degrees. Cross-sectional TEM images show the top layer of GaAs is relatively defect-free and of good crystalline quality. The GaAs layer demonstrates high carrier-mobility and sharp photoluminescence peaks with intensity comparable to GaAs layers grown on rigid, single-crystal substrates. These hetroepitaxial GaAs layers on flexible, large-area, single-crystal-like substrates provide a potential route to fabrication of high-performance devices for optoelectronic applications.

## Data Availability

All data is included in the manuscript.
